# Repeat Traffic Offenders Improve Their Performance in Risky Driving Situations and Have Fewer Accidents Following a Mindfulness-Based Intervention

**DOI:** 10.3389/fpsyg.2020.567278

**Published:** 2021-01-20

**Authors:** Sabina Baltruschat, Laura Mas-Cuesta, Antonio Cándido, Antonio Maldonado, Carmen Verdejo-Lucas, Elvira Catena-Verdejo, Andrés Catena

**Affiliations:** ^1^Mind, Brain and Behavior Research Center (CIMCYC), University of Granada, Granada, Spain; ^2^Presentia, Mindfulness training, Granada, Spain

**Keywords:** mindfulness, risk-taking, repeat traffic offender, emotion regulation, attention regulation

## Abstract

Risky decision-making is highly influenced by emotions and can lead to fatal consequences. Attempts to reduce risk-taking include the use of mindfulness-based interventions (MBI), which have shown promising results for both emotion regulation (ER) and risk-taking. However, it is still unclear whether improved emotion regulation is the mechanism responsible for reduced risk-taking. In the present study, we explore the effect of a 5-week MBI on risky driving in a group of repeat traffic offenders by comparing them with non-repeat offenders and repeat offenders without training. We evaluated the driving behavior of the participants through a driving simulation, and self-reported emotion regulation, both before and after the intervention. At baseline, poor emotion regulation was related to a more unstable driving behavior, and speeding. The group that received mindfulness training showed improved performance during risky driving situations and had fewer accidents, although their overall driving behavior remained largely unchanged. The observed trend toward improved emotion regulation was not significant. We discuss whether other effects of MBI – such as self-regulation of attention – could underlie the observed reduction in risky driving in the initial stages. Nonetheless, our findings still confirm the close relationship between emotion regulation skills and risky driving.

## Introduction

Daily life involves constant decision-making with regard to what actions to take and some situations can lead us to take certain risks, e.g., when we are in a rush or in a bad or even euphoric mood. In fact, the factors that modulate the process of risky decision making include emotion (Angie et al., 2011; Engelmann and Hare, 2018), impulsivity (Nagin and Pogarsky, 2003), and self-regulation (Kelley et al., 2015). Driving is an example of a typical everyday risk-taking scenario, where the most extreme consequences are fatal accidents, estimated at 1.35 million deaths each year (World Health Organization, 2018). Careless driving behavior has been identified as one of the reasons why people take more risks at the wheel and suffer more road accidents (Taubman – Ben-Ari et al., 2016), while speeding has also been linked to higher accident and fatality rates (OECD, 2018).

In driving environments in particular, risky driving and low perception of risk have been found to be influenced by emotions (Nesbit et al., 2007; Megías et al., 2011, 2014; Jeon and Zhang, 2013) and emotion regulation (ER; Navon and Taubman – Ben-Ari, 2019), where ER represents the ability to recognize one’s own emotions and to know how to express and experience them in an adaptive and flexible way (Gratz and Roemer, 2004). Several studies have found that the use of appropriate ER strategies is associated with a safer driving behavior, while difficulties in ER, such as not being aware or able to control impulsive behavior or emotional responses, has been linked to risky driving behavior and traffic violations, for instance exceeding speed limits and mobile use while driving (Hancock et al., 2012; Trógolo et al., 2014; Sani et al., 2017; Šeibokaitė et al., 2017; Parlangeli et al., 2018; Navon and Taubman – Ben-Ari, 2019).

Due to the influence of ER on risky driving, one promising strategy for reducing road fatalities could be the use of interventions that, through the improvement of ER skills, lead to safer driving behavior (Feldman et al., 2011; Koppel et al., 2019). One way of enhancing these skills could be the use of mindfulness-based interventions (MBI), which have been found to produce an improvement in ER (Eberth and Sedlmeier, 2012), even in clinical populations with ER difficulties (Garland et al., 2017; Vanzhula and Levinson, 2020). Mindfulness can be defined as the act of deliberately paying attention to the present moment, with acceptance, openness, and without judgment (Kabat-Zinn, 2005). This deliberate attention involves self-regulation of attention (Tang et al., 2007, 2015; Gil-Jardiné et al., 2017), a fundamental process for the adaptive execution of driving, where attention regulation is essential (Groeger, 2001).

Mindfulness, understood as a trait, is the natural mindful tendency of each individual (Brown and Ryan, 2003) and has been negatively related to risky driving (Feldman et al., 2011; Panek et al., 2015; Koppel et al., 2018; Murphy and Matvienko-Sikar, 2019). In a review of effective interventions for reducing driving anger, Deffenbacher (2016) concluded that MBI reduced anger and facilitated more adaptive expression of anger in drivers (Diebold, 2003; Kazemeini et al., 2013). Some studies have also found that MBIs improve performance on driving simulators, although methodological limitations, such as a very low number of participants and the lack of baseline measurements as in Kass et al. (2011), or the application of only a 10 min mindfulness meditation as in Reynaud and Navarro (2019), and the scarcity of research in this field mean that no firm conclusions can yet be drawn on this issue (for a review, see Koppel et al., 2019).

Thus, in the light of these findings, we aimed to test the effectiveness of a MBI in reducing risky driving behavior in a group of repeat traffic offenders, measuring behavioral change through a driving simulation. This type of measurement has been used to study real driving behavior, with a correspondence between real and simulated driving (Meuleners and Fraser, 2015; Branzi et al., 2017). Specifically, the Honda Riding Trainer (HRT) simulator, used in the present work, has been used to study the processes underlying driving skills (Di Stasi et al., 2010, 2011) and for training in safer driving (Tagliabue et al., 2019). Furthermore, we hypothesized, based on previous research (Koppel et al., 2019), that ER skills would improve and that safer driving is encouraged through improved ER.

## Materials and Methods

### Participants

Our sample was composed of 89 participants (29 women; age range between 18 and 63 years, *M* = 34.39, *SD* = 14.57) recruited in an online survey from the University of Granada (students, teachers, and administration staff), as well as during a rehabilitation course run by a driving school, where drivers recover their points lost because of traffic rule violations. All participants were drivers with a valid driving license. The greater number of men represents the gender differences in driving violations present in the population (Health and Safety Executive, 2002; World Health Organization, 2020).

To group drivers into repeat and non-repeat offenders, we used the following self-reported traffic violations as criteria: attendance of a rehabilitation course for drivers at least once, a loss of points according to the Spanish penalty system for traffic rule violations, being fined at least twice for risky driving behavior (alcohol or drug use, not using a seat belt, or exceeding speed limits), or reporting as having usually exceeded speed limits by more than 20% of the permitted speed. Sixty repeat offenders, meeting at least one of these criteria, and 29 non-repeat offenders, meeting none of these criteria, completed the baseline and post-intervention evaluations.

Half of the risky drivers were selected for a 5-week MBI program dependent on their weekly availability, which was established prior to testing, to gather the greatest number of participants for the intervention groups. At four different time points along the 2-year period of data collection, we grouped the participants who coincided at the same weekday availability, resulting in a quasi-randomized controlled trial. The drop-out rate of the mindfulness training following the second session was 6 out of the 30 participants.

Thus, in the current study, we compared the following three groups: non-repeat offenders (NR, *N* = 29), repeat offenders (R, *N* = 30), and repeat offenders who received mindfulness training (R-M, *N* = 30; see Table 1 for more details).

**Table 1:** Demographic variables and driving experience (mean and standard deviation).

	NR	R	R-M
Age	32.38 (14.6)	35.03 (14.66)	35.7 (14.75)
Sex (women/men)	11/18	6/24	12/18
Education level	3.55^*^(0.51)	3.67^*^(0.61)	3.4^*^(0.62)
Estimated km driven in life	228867.24 (424848.94)	324144.83 (401172.55)	373083.33 (606785.13)
Interval of estimated km driven per year by car	5.1^**^(2.82)	6.59^**^(3.21)	5.83^**^(3)

* Education ranged from 2 (Primary studies) to 4 (Superior level studies).

** Km driven/years is measured in estimated intervals, with 5 = 6,000-9,000; 6 = 9,000-12,000; and 7 = 12,000–15,000 km.

### Questionnaires

We used two complementary questionnaires, focusing on the awareness of emotion and its regulation and different types of ER strategies, respectively.

#### Self-Reported Traffic Violation

To group participants into repeat and non-repeat offenders they reported on demographic variables (sex and age), km driven per year and in life, months of holding a driver license, number of rehabilitation courses, number of lost points, number of traffic fines, and frequency of exceeding speed limits.

#### Difficulties of Emotion Regulation Scale

The Spanish version of the Difficulties Emotion Regulation Scale (DERS; Gratz and Roemer, 2004; Gómez-Simón et al., 2014) measures different negative aspects of emotion recognition, control, and regulation strategies. The 36-item questionnaire consists of six subscales, using a five-point Likert scale from 1 (Almost never, 0–10%) to 5 (Almost always, 91–100%) evaluates the following: *Lack of emotional awareness* (six items), *Impulse control difficulties* (six items), *Non-acceptance of emotional response* (seven items), *Difficulties engaging in goal-directed behavior* (five items), *Lack of emotional clarity* (five items), and *Limited access to emotion regulation strategies* (seven items). The internal consistency of the scale is adequate (Cronbach’s *α* = 0.88).

#### Cognitive Emotion Regulation Questionnaire

The Spanish version of the Cognitive Emotion Regulation Questionnaire (CERQ; Garnefski et al., 2001; Domínguez-Sánchez et al., 2013) measures different types of ER strategies. The 36-item questionnaire consists of nine subscales with four items each, using the same five-point Likert as the DERS: *Self-Blame* (Cronbach’s *α* = 0.61), *Rumination* (Cronbach’s *α* = 0.74), *Catastrophizing* (Cronbach’s *α* = 0.72), *Other-Blame* (Cronbach’s *α* = 0.79), *Acceptance* (Cronbach’s *α* = 0.64), *Positive reinforcing* (Cronbach’s *α* = 0.89), *Refocus on planning* (Cronbach’s *α* = 0.83), *Positive reappraisal* (Cronbach’s *α* = 0.8), and *Putting into perspective* (Cronbach’s *α* = 0.83). The first four and the remaining five subscales were grouped into negative and positive ER strategies (Cronbach’s *α* = 0.89/0.79, respectively), respectively, as suggested by the original authors (Garnefski et al., 2001).

### Driving Simulation

For the driving simulation, we used the HRT motorcycle simulator, which consists of a seat, handlebar, pedals, accelerator, brakes, turn indicators, and claxon (see Di Stasi et al., 2009; Megías et al., 2017, for more details). All participants rode through the same three different traffic scenarios in a counterbalanced order to measure driving behavior in different contexts: two urban scenarios, one by day and the other by night, and a mountain road scenario. Each of these scenarios contained a total of eight risk situations, such as crossing pedestrians or obstacles on the road, and was approximately 5–10 min long depending on the type of scenario, speed, crashes, and variability of the course taken by the participant. They were projected on the wall in front of the participants seated on the motorcycle simulator at a distance of 185 cm on a 110 × 180 cm screen, with a refresh rate of 30 Hz and a resolution of 1,024 × 768 pixels.

Indices calculated from data recorded by the HRT included: average and variance of speed (km/h), of speed in a risk situation (km/h), and of exceeded speed limits (km/h), length of time spent exceeding speed limits (sec), average throttle rotation (%), variance of steering wheel (rad), front and rear brake force (kg), number of accidents, and the average rating of performance in each risk situation, calculated by the HRT, ranging from A (good performance) to D (accident), taking into account variables, such as speed when entering the risk situation and distance to crash with an object. This last value is coded from 1 to 4 with lower values indicating greater risk-taking and worse performance in a risk situation. Exceeded speed limits, speed in risk situations, and performance ratings are calculated for the two urban courses only, since the HRT software does not register measures of speed limits or risk situations in the mountain road scenario.

### Mindfulness-Based Training

The mindfulness-based training was adapted from the Mindfulness-Based Stress Reduction (MBSR) program widely used in research (Kabat-Zinn, 2003). The program length was reduced to 5 weeks with 3-h weekly sessions due to the availability of the participants. The sessions were prepared by an instructor with extensive experience in mindfulness-based training and were delivered by the instructor herself (CVL) and another instructor (ECV) with experience in the MBSR program. To avoid the influence of researcher bias, neither of the instructors was involved in the data collection. The sessions were designed to enhance situation awareness and included meditation and yoga practice (attention to breathing, body scanning, yoga, and guided meditation), group discussions, and training in ER, as well as the importance of focusing on what happens in the present moment both inside and outside, and pausing to take a breath before observing and finally selecting the appropriate response.

### Procedure

The participants were selected according to their self-reported traffic violations, which were requested by means of an online survey (see participants section). The baseline and post-intervention evaluation was the same. Participants came to the research center and, as a part of a broader project, filled in the questionnaires and completed the driving simulation, with the order based on the availability of the facilities (HRT and computer room) and participants’ temporal availability. The average interval between both evaluations was approximately 4 months (Mean = 142.26 days, *SD* = 69.15) and varied across the participants due to their availability. Therefore, the length of this interval was included as a factor in the data analysis.

### Data Analysis

As in the literature recommended, we used an intention-to-treat approach (Gupta, 2011), analyzing participants who dropped-out in the R-M group.

JASP statistical software (Version 0.11.1, JASP Team, 2020, freely available at https://jasp-stats.org/) and R Studio (RStudio: Integrated Development for RStudio, Inc., Boston, MA, 2016, freely available at http://www.rstudio.com/) were used for analyses, along with *p* value null hypothesis statistical testing (NHST) and Bayesian methods.

We found no differences between the three groups in terms of gender (Pearson’s *χ^2^* = 3.291, *df* = 2, *p* = 0.193), education level (Kruskal Wallis *H* = 0.832, *df* = 2, *p* = 0.66), age [*F*(2, 86) = 0.421, *p* = 0.658], or driving experience [km driven in life: *F*(2, 85) = 0.664, *p* = 0.518; km/year by car: *F*(2, 85) = 1.751, *p* = 0.18].

Mixed-factor ANCOVAS were conducted to analyze the effect of the intervention on the driving behavior indices and the ER strategies. The experimental design includes time (baseline and post-intervention evaluation) and subscales as within-subject factors, and group (NR, R, and R-M) as between-subject factors, using age and interval between evaluations as covariates. To support our hypothesis with Bayesian methods, the Bayes Factor (BF_10_) was calculated for all possible models compared to the null model, as well as the BF_Inclusion_ for each predictor (Wagenmakers et al., 2018; Bergh et al., 2020), estimated across all matched models following Sebastiaan Mathôd (Bergh et al., 2020).

For variables with significant time × group interactions, mediation analysis was conducted. To explore the magnitude of the intervention effect, an ANCOVA was carried out on the Behavior Shift Index (BSI; Li et al., 2018), defined as the magnitude of change between baseline and post-training evaluation [(Value_baseline_ – Value_post-training_)/(Value_baseline_ + Value_post-training_)] with group as the between-subjects factor and age and interval between evaluations as covariates.

As accident rate is not a continuous variable, these rates are analyzed by categorizing the difference between baseline and post-intervention evaluation into improved (difference > 0), unchanged (difference = 0), and worse performance (difference < 0). A multinomial regression was conducted, and risk ratios were estimated with group (NR, R, and R-M) as the between-subjects factor and age and interval between evaluations as covariates.

Finally, we explored whether ER and driving behavior are related, conducting a partial correlation analysis between the BSI values of all questionnaire subscales and the driving behavior indices, using age as a covariate. We also confirmed a general relationship between ER and the driving behavior indices at baseline in the whole sample (Supplementary Material).

## Results

### Effect of the Intervention on Emotion Regulation and Driving Behavior

**Figure 1:**
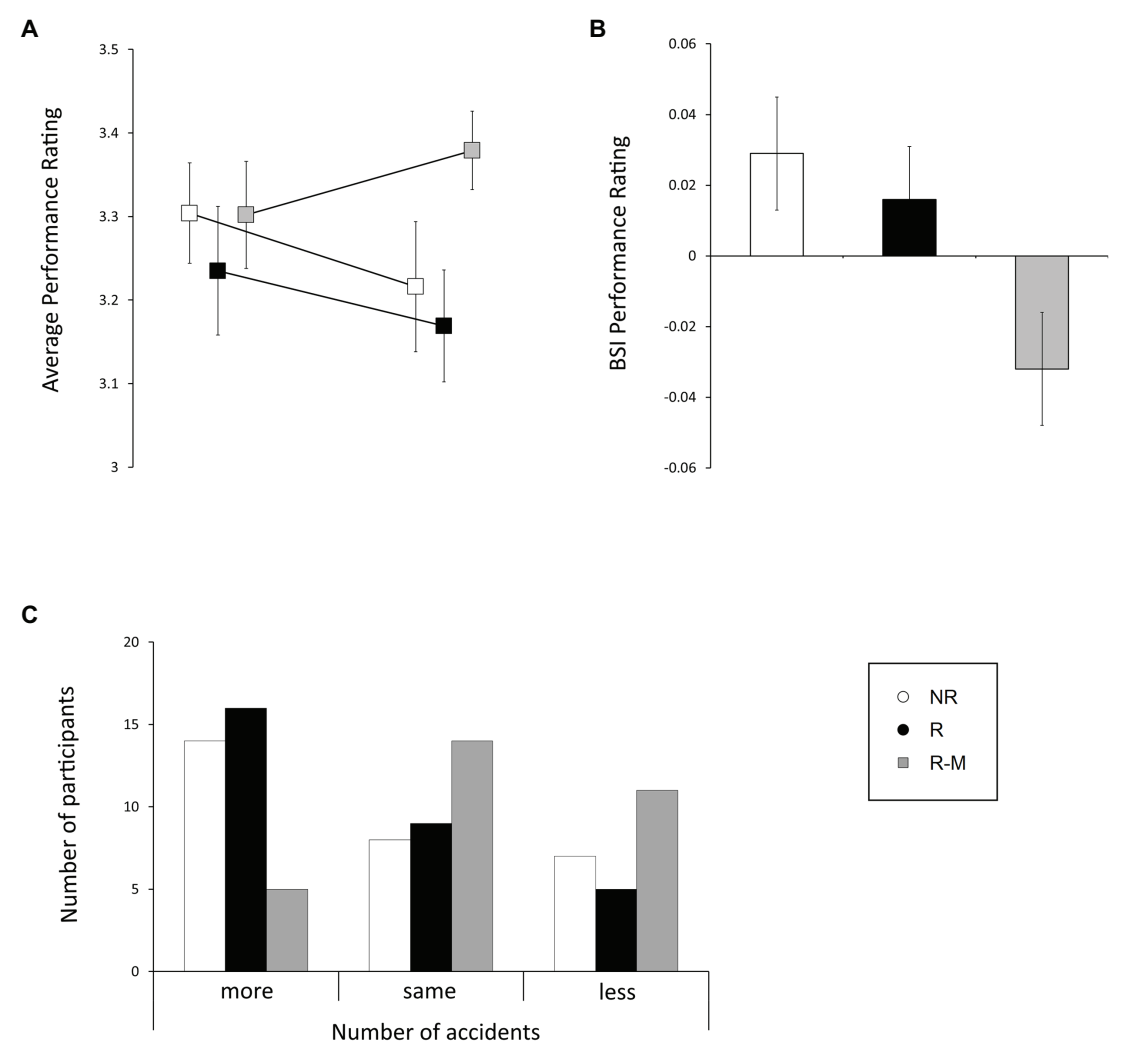
Effect of the intervention on the behavioral indices of the driving simulation. **(A)** represents the differences in average evaluation between baseline and post-intervention, **(B)** indicates the magnitude of behavioral changes in the average performance ratings in risk situations in each group, and **(C)** shows the differences in the number of accidents between baseline and post-intervention.

Analysis of the driving performance ratings revealed a time × group interaction [*F*(2, 84) = 3.919, *p* = 0.024, *η*^2^ = 0.085, BF_Inclusion_ = 2.255], as well as a main effect of age [*F*(1, 84) = 24.798, *p* < 0.001, *η^2^* = 0.228, BF_Inclusion_ = 4166.733, best model BF_10_ = 4503.795; Figure 1A]. Mediation analysis revealed differences between baseline and post-intervention evaluation in the R-M group [*F*(1) = 10.642, *p* = 0.003] and no differences between the two control groups (*p* > 0.4). The ANCOVA on BSI scores, indicating the magnitude of changes, revealed a main effect of group [*F*(2, 84) = 4.062, *p* = 0.021, *η^2^* = 0.088, BF_Inclusion_ = 1.993] and no main effect of age [*F*(2, 84) = 2.869, *p* = 0.094, *η^2^* = 0.033, BF_Inclusion_ = 0.57; Figure 1B].

No significant time × group interaction effects (*p* > 0.1) were found for the remaining indices, although a significant main effect of age was found (*p* < 0.001). However, since the performance rating is based on other indices during the risk situations, we observed strong associations between all of these indices and the baseline performance ratings (Pearson’s *r*: min = −0.338, *p* < 0.001, max = −0.847, *p* < 0.001; Table 2), while the improvement in performance rating measured with the BSI is also associated with the BSI of the other indices (Pearson’s *r*: min = −0.253, *p* < 0.05, max = −0.624, *p* < 0.001; Table 2), which is even stronger and more consistent in the R-M group (Pearson’s *r*: min = −0.407, *p* < 0.05, max = −0.835, *p* < 0.001; Table 2).

**Table 2:** Relationship between evaluation of performance in risk situations, as calculated by the Honda Riding Trainer (HRT), and other HRT indices.

	Average speed (km/h)	Variance speed (km/h)	Average speed risk situations (km/h)	Variance speed risk situations (km/h)	Average exceeded speed limits (km/h)	Variance exceeded speed limits (km/h)	Length of time spent exceeding speed (s)	Mean throttle (%)	Front brake (kg)	Variance steering wheel (rad)	Accidents (sum)
Evaluation of performance in risk situation (1–4)	−0.752^**^	−0.549^**^	−0.847^**^	−0.611^**^	−0.642^**^	−0.528^**^	−0.762^**^	−0.563^**^	−0.338^**^	−0.691^**^	−0.473^**^
Correlations of BSI values	−0.464^**^	−0.26^*^	−0.624^**^	−0.313^*^	−0.184	−0.219^*^	−0.253^*^	−0.273^*^	−0.082	−0.434^**^	—
Correlations of BSI in the R-M group	−0.685^**^	−0.453^*^	−0.835^**^	−0.542^**^	−0.231	−0.317	−0.438^*^	−0.407^*^	−0.322	−0.528^**^	—

** *p* < 0.001;

* *p* < 0.05.

Rear Brake Index did not show any significant relationship. Behavior Shift Index (BSI) for accident rate could not be calculated as variable is not continues. Partial correlation coefficient reported represent Pearson’s *r*, except for accident rates where Spearman’s rho is reported.

The multinomial regression analysis of the differences in accident numbers between baseline and post-intervention evaluations revealed differences between the R-M and both control groups in the comparison between worse and better performance (*AIC* = 196.67, R-M vs. R: 1.851, *p* = 0.016, 95% CI = 0.347–3.355; R-M vs. NR: 1.464, *p* = 0.046, 95% CI = 0.029–2.9), while the control groups did not differ from each other (R vs. NR: −0.387, *p* = 0.58, 95% CI = −1.756–0.982; Figure 1C). Comparing the outcomes of more and fewer accidents using the relative risk ratios, R are 6.368 times, and NR are 4.325 times, more likely to have more accidents compared with the M-R group.

**Figure 2:**
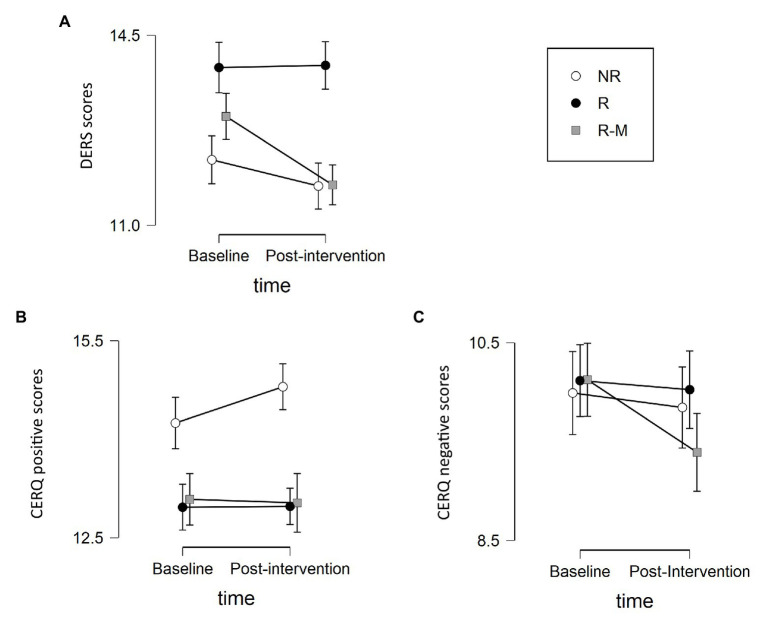
Differences in the emotion regulation (ER) scales between the baseline and post-intervention for each group. Total scores are presented for the Difficulties Emotion Regulation Scale (DERS; **A**) and the positive **(B)** and negative **(C)** scales of the Cognitive Emotion Regulation Questionnaire (CERQ).

No time × group interaction effect was found for ER [DERS: *F*(2, 84) = 2.264, *p* = 0.11, *η^2^* = 0.051, BF_Inclusion_ = 0.396; CERQ positive: *F*(2, 84) = 0.788, *p* = 0.458, *η^2^* = 0.018, BF_Inclusion_ = 0.07; and CERQ negative: *F*(2, 84) = 1.451, *p* = 0.24, *η^2^* = 0.033, BF_Inclusion_ = 0.091]. However, there was a tendency for the R-M group to show improvement in the DERS and the negative scales of the CERQ, which can be observed in Figure 2.

In summary, participants who were trained in mindfulness do not show differences in ER but showed improved performance in risk situations and had fewer accidents in comparison with both control groups. It is also worth noting that while age is an important factor in the prediction of driving behavior, this factor has almost no influence on the magnitude of improvement observed as a consequence of the intervention.

## Discussion

In the current study, we explored the effect of MBI on driving behavior and ER. We evaluated the performance on a driving simulation and self-reported ER scores of a group of repeat offenders trained in mindfulness and compared these measures with those of two control groups, one of repeat offenders and another of non-repeat offenders. We found that the intervention had an effect on accidents and evaluation of performance in risk situations, but no effect on ER and most of the behavioral indices. However, driving indices were closely related to the performance ratings at baseline, while the magnitude of change was related to the one of performance ratings, being greatest in the mindfulness trained group.

### Effect of Intervention on Driving Behavior

The R-M group of our study had fewer accidents and performed better in a risk situation, although no differences were found in terms of the other driving indices, such as speed, acceleration, and driving direction. However, these indices are closely related to performance ratings in risk situations, and the magnitude of change observed in these measures is strongly associated with the change in performance ratings in the R-M group, pointing to the possibility that most of the indices are enhanced in a similar way. As mentioned earlier, the length of the intervention could have played a role in the non-significance of some of these effects, which might be greater in a follow-up study.

Previous research on the effect of mindfulness training on driving behavior is still scarce. In fact, there is only one study exploring changes in driving simulation, which found a (non-significant) reduction in traffic violations in students enrolled in a Buddhism course (Kass et al., 2011). Since these authors reported high correlations between situation awareness of the driving simulation and the scores in mindfulness and concentration, the mechanism of driving improvements through meditation training might be due to the greater attention paid to risk factors on the road (Kass et al., 2011), which would also be consistent with our results.

Furthermore, our findings showed that age is one of the most important predictors of risky driving behavior on the simulator, characterized by speeding, an instable direction, and low evaluations in the performance in risk situations, which is in line with previous research (Jonah, 1990; Rhodes and Pivik, 2011). However, we did not find an effect of age on the magnitude of change produced by the intervention, which suggests that MBI is equally beneficial for all age groups. This could have practical implications for the design of new intervention programs that are primarily aimed at youths and novice drivers, who are the groups that suffer the most fatalities (World Health Organization, 2018).

### Effect of the Intervention on Emotion Regulation

In the present study, we found no differences in ER following the 5-week MBI, although the pattern of results points to less ER difficulties and a reduction of negative ER strategies, such as ruminations, catastrophizing, and blaming oneself or others. Studies with a longer intervention – usually 8 weeks – have found enhanced ER (for a review, see Roemer et al., 2015). Thus, the lack of significant findings reported here could be due to the length of the intervention, since, even after 5 weeks, we were already able to observe some effects of the intervention.

In the driving context, research has focused on driving anger and aggressive driving, applying a wide variety of approaches, including behavioral, cognitive, and relaxation techniques (for a review, see Deffenbacher, 2016). The first studies to apply MBI found improvements in driving anger (Diebold, 2003; Kazemeini et al., 2013). However, methodological limitations, such as small sample sizes and the sole use of questionnaires make it difficult to draw any firm conclusions.

Moreover, training in mindfulness may not only reduce driving anger, but might also produce other changes in ER strategies that affect aberrant driving behavior. Thus, it is important to measure changes in emotion regulation or expression in general. This issue was addressed in a study with Chinese bus drivers, where cognitive therapy, using the same type of ER instruction as that used in the present intervention, resulted in the greater use of positive ER strategies (Feng et al., 2018). These findings may help to explain the differences we found in the observed changes associated with the distinct ER strategies. Positive regulation strategies, such as positive reappraisal, may be enhanced by the components of cognitive therapy used in the MBI. On the other hand, mindfulness meditation itself could enhance ER processes, such as emotional awareness and clarity, impulsive control, and acceptance of emotional responses, as well as reduce negative ER strategies. This would be in line with neuroscientific approaches, where two different mechanisms have been suggested for the enhancement of ER through mindfulness top-down and bottom-up processes (Chiesa et al., 2013; Guendelman et al., 2017).

### Emotion Regulation as a Mechanism of Improvement

Taken together, our results provide first evidence of a behavioral change following MBI in repeat offenders, a high-risk group for road accidents and fatalities. Since in the current study, behavior is measured in a simulated traffic environment, and not only with questionnaires or decision-making tasks, the results are promising and suggestive of real-life behavior. Additionally, it should be noted that, even though a motorcycle simulator was used, these results may indicate safer driving behavior in general, as well in other vehicles such as cars and bikes.

Although research has pointed to ER as the mechanism underlying safer driving behavior (Feldman et al., 2011; Koppel et al., 2019), our results only indicate a (non-significant) tendency for less ER difficulties and the use of fewer negative ER strategies, such as rumination, catastrophizing, and self- and other-blame.

We hypothesize that the first behavioral changes may be faster and easier to measure than differences in ER, which may be more stable over time. The improvements in attentional control, which are enhanced by MBI (Tang et al., 2007; Malinowski, 2013), might be greater in these first weeks, generating better performance in risk situations, and thereby leading to fewer accidents. By paying more attention to road signals, conditions, and signs of risk, they may have improved risk perception, and thus, drive safely (Kass et al., 2011). Changes in ER could require more practice and thus may not be directly responsible for the behavioral changes we found in the present study. Nonetheless, our baseline correlations between ER and driving indices of the driving simulation (Supplementary Table 1) suggest an association between ER and driving behavior. Therefore, more research is needed to identify the precise mechanism by which mindfulness training can enhance safe driving behavior.

### Limitations

Although our findings indicate that MBI lead to a safer performance in risk situation, more research is needed to confirm our results and to study long-term effect. Since our sampling was based on the temporal availability of the participants, complete randomized trials are needed with a greater number of participants, as well as studies using longer MBI programs, to explore whether longer training improves ER and other indices of driving behavior.

## Data Availability Statement

The raw data supporting the conclusions of this article will be made available by the authors, without undue reservation, to any qualified researcher.

## Ethics Statement

The studies involving human participants were reviewed and approved by the Human Research Ethics Committee of the University of Granada (n° 204/CEIH/2016). The patients/participants provided their written informed consent to participate in this study.

## Author Contributions

ACt, ACn, AM, and SB designed the experiment. LM-C and SB carried out the testing of participants. CV-L and EC-V designed and performed the intervention. SB and ACt analyzed the data. SB and LM-C drafted the manuscript. All authors contributed to the critical revision of the manuscript and approved the final version.

### Conflict of Interest

CV-L and EC-V were employed by the company Presentia.

The remaining authors declare that the research was conducted in the absence of any commercial or financial relationships that could be construed as a potential conflict of interest.

## References

[ref1] Angie A. D. Connelly S. Waples E. P. Kligyte V. (2011). The influence of discrete emotions on judgement and decision-making: a meta-analytic review. Cognit. Emot. 25, 1393–1422. 10.1080/02699931.2010.550751, PMID: 21500048

[ref3] Bergh D. van den Doorn J. van Marsman M. Draws T. Kesteren E. -J. van Derks K. (2020). A tutorial on conducting and interpreting a Bayesian ANOVA in JASP. L’Année Psychol. 120, 73–96. 10.3917/anpsy1.201.0073

[ref4] Branzi V. Domenichini L. La Torre F. (2017). Drivers’ speed behaviour in real and simulated urban roads—a validation study. Transp. Res. Part F Traffic Psychol. Behav. 49, 1–17. 10.1016/j.trf.2017.06.001

[ref5] Brown K. W. Ryan R. M. (2003). The benefits of being present: mindfulness and its role in psychological well-being. J. Pers. Soc. Psychol. 84, 822–848. 10.1037/0022-3514.84.4.822, PMID: 12703651

[ref6] Chiesa A. Serretti A. Jakobsen J. C. (2013). Mindfulness: top–down or bottom–up emotion regulation strategy? Clin. Psychol. Rev. 33, 82–96. 10.1016/j.cpr.2012.10.006, PMID: 23142788

[ref7] Deffenbacher J. L. (2016). A review of interventions for the reduction of driving anger. Transp. Res. Part F Traffic Psychol. Behav. 42, 411–421. 10.1016/j.trf.2015.10.024

[ref8] Di Stasi L. L. Álvarez-Valbuena V. Cañas J. J. Maldonado A. Catena A. Antolí A. (2009). Risk behaviour and mental workload: multimodal assessment techniques applied to motorbike riding simulation. Transp. Res. Part F Traffic Psychol. Behav. 12, 361–370. 10.1016/j.trf.2009.02.004

[ref9] Di Stasi L. L. Contreras D. Cañas J. J. Cándido A. Maldonado A. Catena A. (2010). The consequences of unexpected emotional sounds on driving behaviour in risky situations. Saf. Sci. 48, 1463–1468. 10.1016/j.ssci.2010.07.006

[ref10] Di Stasi L. L. Contreras D. Cándido A. Cañas J. J. Catena A. (2011). Behavioral and eye-movement measures to track improvements in driving skills of vulnerable road users: first-time motorcycle riders. Transp. Res. Part F Traffic Psychol. Behav. 14, 26–35. 10.1016/j.trf.2010.09.003

[ref11] Diebold J. C. (2003). Mindfulness in the machine: A mindfulness-based cognitive therapy for the reduction of driving anger. Available at: https://search.proquest.com/docview/304625552/abstract/8C5DA2C0F45A44E8PQ/1 (Accessed May 11, 2020).

[ref12] Domínguez-Sánchez F. J. Lasa-Aristu A. Amor P. J. Holgado-Tello F. P. (2013). Psychometric properties of the Spanish version of the cognitive emotion regulation questionnaire. Assessment 20, 253–261. 10.1177/1073191110397274, PMID: 21467092

[ref13] Eberth J. Sedlmeier P. (2012). The effects of mindfulness meditation: a meta-analysis. Mindfulness 3, 174–189. 10.1007/s12671-012-0101-x

[ref14] Engelmann J. B. Hare T. A. (2018). “Emotions can bias decision-making processes by promoting specific behavioral tendencies” in The nature of emotion: Fundamental questions. eds. Fox A. S. Lapate R. C. Shackman A. J. Davidson R. J.(New York: Oxford University Press), 355–359.

[ref15] Feldman G. Greeson J. Renna M. Robbins-Monteith K. (2011). Mindfulness predicts less texting while driving among young adults: examining attention- and emotion-regulation motives as potential mediators. Personal. Individ. Differ. 51, 856–861. 10.1016/j.paid.2011.07.020, PMID: 22031789 PMC3199141

[ref16] Feng Z. Zhan J. Ma C. Lei Y. Liu J. Zhang W. (2018). Is cognitive intervention or forgiveness intervention more effective for the reduction of driving anger in Chinese bus drivers? Transp. Res. Part F Traffic Psychol. Behav. 55, 101–113. 10.1016/j.trf.2018.02.039

[ref17] Garland E. L. Hanley A. W. Goldin P. R. Gross J. J. (2017). Testing the mindfulness-to-meaning theory: evidence for mindful positive emotion regulation from a reanalysis of longitudinal data. PLoS One 12:e0187727. 10.1371/journal.pone.0187727, PMID: 29211754 PMC5718463

[ref18] Garnefski N. Kraaij V. Spinhoven P. (2001). Negative life events, cognitive emotion regulation and emotional problems. Personal. Individ. Differ. 30, 1311–1327. 10.1016/S0191-8869(00)00113-6

[ref19] Gil-Jardiné C. Née M. Lagarde E. Schooler J. Contrand B. Orriols L.. (2017). The distracted mind on the wheel: overall propensity to mind wandering is associated with road crash responsibility. PLoS One 12:e0181327. 10.1371/journal.pone.0181327, PMID: 28771623 PMC5542598

[ref20] Gómez-Simón I. Penelo E. de la Osa N. (2014). Factor structure and measurement invariance of the difficulties emotion regulation scale (DERS) in Spanish adolescents. Psicothema 26, 401–408. 10.7334/psicothema2013.324, PMID: 25069562

[ref21] Gratz K. L. Roemer L. (2004). Multidimensional assessment of emotion regulation and dysregulation: development, factor structure, and initial validation of the difficulties in emotion regulation scale. J. Psychopathol. Behav. Assess. 26, 41–54. 10.1023/B:JOBA.0000007455.08539.94

[ref22] Groeger J. A. (2001). Understanding driving: Applying cognitive psychology to a complex everyday task. Reprinted. Hove: Psychology Press.

[ref23] Guendelman S. Medeiros S. Rampes H. (2017). Mindfulness and emotion regulation: insights from neurobiological, psychological, and clinical studies. Front. Psychol. 8:220. 10.3389/fpsyg.2017.00220, PMID: 28321194 PMC5337506

[ref24] Gupta S. (2011). Intention-to-treat concept: a review. Perspect. Clin. Res. 2:109. 10.4103/2229-3485.83221, PMID: 21897887 PMC3159210

[ref25] Hancock G. M. Hancock P. A. Janelle C. M. (2012). The impact of emotions and predominant emotion regulation technique on driving performance. Work 41, 3608–3611. 10.3233/WOR-2012-0666-3608, PMID: 22317270

[ref26] Health and Safety Executive (2002). The contribution of individual factors to driving b. Available at: https://www.hse.gov.uk/research/rrhtm/rr020.htm (Accessed July 23, 2020).

[ref27] Jeon M. Zhang W. (2013). Sadder but wiser? Effects of negative emotions on risk perception, driving performance, and perceived workload. Proc. Hum. Factors Ergon. Soc. Annu. Meet. 57, 1849–1853. 10.1177/1541931213571413

[ref28] Jonah B. A. (1990). Age differences in risky driving. Health Educ. Res. 5, 139–149. 10.1093/her/5.2.139

[ref29] Kabat-Zinn J. (2003). Mindfulness-based interventions in context: past, present, and future. Clin. Psychol. Sci. Pract. 10, 144–156. 10.1093/clipsy.bpg016

[ref30] Kabat-Zinn J. (2005). Coming to our senses: Healing ourselves and the world through mindfulness. New York: Hyperion.

[ref31] Kass S. J. VanWormer L. A. Mikulas W. L. Legan S. Bumgarner D. (2011). Effects of mindfulness training on simulated driving: preliminary results. Mindfulness 2, 236–241. 10.1007/s12671-011-0066-1

[ref32] Kazemeini T. Ghanbari-e-Hashem-Abadi B. Safarzadeh A. (2013). Mindfulness based cognitive group therapy vs cognitive behavioral group therapy as a treatment for driving anger and aggression in iranian taxi drivers. Psychology 04, 638–644. 10.4236/psych.2013.48091

[ref33] Kelley W. M. Wagner D. D. Heatherton T. F. (2015). In search of a human self-regulation system. Annu. Rev. Neurosci. 38, 389–411. 10.1146/annurev-neuro-071013-014243, PMID: 25938728 PMC4530781

[ref34] Koppel S. Bugeja L. Hua P. Osborne R. Stephens A. N. Young K. L.. (2019). Do mindfulness interventions improve road safety? A systematic review. Accid. Anal. Prev. 123, 88–98. 10.1016/j.aap.2018.11.013, PMID: 30468950

[ref35] Koppel S. Stephens A. N. Young K. L. Hua P. Chambers R. Hassed C. (2018). What is the relationship between self-reported aberrant driving behaviors, mindfulness, and self-reported crashes and infringements? Traffic Inj. Prev. 19, 480–487. 10.1080/15389588.2018.1440083, PMID: 29580093

[ref36] Li Y. Liu F. Zhang Q. Liu X. Wei P. (2018). The effect of mindfulness training on proactive and reactive cognitive control. Front. Psychol. 9:1002. 10.3389/fpsyg.2018.01002, PMID: 29973897 PMC6019482

[ref37] Malinowski P. (2013). Neural mechanisms of attentional control in mindfulness meditation. Front. Neurosci. 7:8. 10.3389/fnins.2013.00008, PMID: 23382709 PMC3563089

[ref38] Megías A. Cortes A. Maldonado A. Cándido A. (2017). Using negative emotional feedback to modify risky behavior of young moped riders. Traffic Inj. Prev. 18, 351–356. 10.1080/15389588.2016.1205189, PMID: 27580253

[ref39] Megías A. Di Stasi L. L. Maldonado A. Catena A. Cándido A. (2014). Emotion-laden stimuli influence our reactions to traffic lights. Transp. Res. Part F Traffic Psychol. Behav. 22, 96–103. 10.1016/j.trf.2013.09.017

[ref40] Megías A. Maldonado A. Cándido A. Catena A. (2011). Emotional modulation of urgent and evaluative behaviors in risky driving scenarios. Accid. Anal. Prev. 43, 813–817. 10.1016/j.aap.2010.10.029, PMID: 21376870

[ref41] Meuleners L. Fraser M. (2015). A validation study of driving errors using a driving simulator. Transp. Res. Part F Traffic Psychol. Behav. 29, 14–21. 10.1016/j.trf.2014.11.009

[ref42] Murphy G. Matvienko-Sikar K. (2019). Trait mindfulness & self-reported driving behaviour. Personal. Individ. Differ. 147, 250–255. 10.1016/j.paid.2019.05.002

[ref43] Nagin D. Pogarsky G. (2003). An experimental investigation of deterrence: cheating self-serving bias, and impulsivity. Criminology 41, 167–194. 10.1111/j.1745-9125.2003.tb00985.x

[ref44] Navon M. Taubman – Ben-Ari O. (2019). Driven by emotions: the association between emotion regulation, forgivingness, and driving styles. Transp. Res. Part F Traffic Psychol. Behav. 65, 1–9. 10.1016/j.trf.2019.07.005

[ref45] Nesbit S. M. Conger J. C. Conger A. J. (2007). A quantitative review of the relationship between anger and aggressive driving. Aggress. Violent Behav. 12, 156–176. 10.1016/j.avb.2006.09.003

[ref46] OECD (2018). Speed and Crash Risk. Available at: https://www.itf-oecd.org/speed-crash-risk (Accessed May 16, 2020).

[ref47] Panek E. T. Bayer J. B. Dal Cin S. Campbell S. W. (2015). Automaticity, mindfulness, and self-control as predictors of dangerous texting behavior. Mob. Media Commun. 3, 383–400. 10.1177/2050157915576046

[ref48] Parlangeli O. Bracci M. Guidi S. Marchigiani E. Duguid A. M. (2018). Risk perception and emotions regulation strategies in driving behaviour: an analysis of the self-reported data of adolescents and young adults. Int. J. Hum. Factors Ergon. 5:166. 10.1504/IJHFE.2018.092242

[ref49] Reynaud E. Navarro J. (2019). “Chapter 15 – is mindfulness helping the brain to drive? Insights from behavioral data and future directions for research” in Neuroergonomics. eds. Ayaz H. Dehais F. (Academic Press), 93–97.

[ref50] Rhodes N. Pivik K. (2011). Age and gender differences in risky driving: the roles of positive affect and risk perception. Accid. Anal. Prev. 43, 923–931. 10.1016/j.aap.2010.11.015, PMID: 21376884

[ref51] Roemer L. Williston S. K. Rollins L. G. (2015). Mindfulness and emotion regulation. Curr. Opin. Psychol. 3, 52–57. 10.1016/j.copsyc.2015.02.006

[ref52] Sani S. R. H. Tabibi Z. Fadardi J. S. Stavrinos D. (2017). Aggression, emotional self-regulation, attentional bias, and cognitive inhibition predict risky driving behavior. Accid. Anal. Prev. 109, 78–88. 10.1016/j.aap.2017.10.006, PMID: 29049929

[ref53] Šeibokaitė L. Endriulaitienė A. Sullman M. J. M. Markšaitytė R. Žardeckaitė-Matulaitienė K. (2017). Difficulties in emotion regulation and risky driving among Lithuanian drivers. Traffic Inj. Prev. 18, 688–693. 10.1080/15389588.2017.1315109, PMID: 28384034

[ref54] Tagliabue M. Sarlo M. Gianfranchi E. (2019). How can on-road hazard perception and anticipation be improved? Evidence from the body. Front. Psychol. 10:167. 10.3389/fpsyg.2019.00167, PMID: 30774617 PMC6367247

[ref55] Tang Y. -Y. Hölzel B. K. Posner M. I. (2015). The neuroscience of mindfulness meditation. Nat. Rev. Neurosci. 16, 213–225. 10.1038/nrn3916, PMID: 25783612

[ref56] Tang Y. -Y. Ma Y. Wang J. Fan Y. Feng S. Lu Q.. (2007). Short-term meditation training improves attention and self-regulation. Proc. Natl. Acad. Sci. U. S. A. 104, 17152–17156. 10.1073/pnas.0707678104., PMID: 17940025 PMC2040428

[ref57] Taubman – Ben-Ari O. Kaplan S. Lotan T. Prato C. G. (2016). The combined contribution of personality, family traits, and reckless driving intentions to young men’s risky driving: what role does anger play? Transp. Res. Part F Traffic Psychol. Behav. 42, 299–306. 10.1016/j.trf.2015.10.025

[ref58] Trógolo M. A. Melchior F. Medrano L. A. (2014). The role of difficulties in emotion regulation on driving behavior. J. Behav. Health Soc. Issues 6, 107–117. 10.5460/jbhsi.v6.1.47607

[ref59] Vanzhula I. A. Levinson C. A. (2020). Mindfulness in the treatment of eating disorders: theoretical rationale and hypothesized mechanisms of action. Mindfulness 11, 1090–1104. 10.1007/s12671-020-01343-4

[ref60] Wagenmakers E. -J. Marsman M. Jamil T. Ly A. Verhagen J. Love J.. (2018). Bayesian inference for psychology. Part I: theoretical advantages and practical ramifications. Psychon. Bull. Rev. 25, 35–57. 10.3758/s13423-017-1343-3, PMID: 28779455 PMC5862936

[ref61] World Health Organization (2018). Global status report on road safety 2018. Available at: https://www.who.int/publications-detail/global-status-report-on-road-safety-2018 (Accessed May 16, 2020).

[ref62] World Health Organization (2020). Road traffic injuries. Available at: https://www.who.int/news-room/fact-sheets/detail/road-traffic-injuries (Accessed July 23, 2020).

