# Supplementary material for: Repeat Traffic Offenders Improve Their Performance in Risky Driving Situations and Have Fewer Accidents Following a Mindfulness-Based Intervention
Source: Front Psychol. 2021 Jan 20;11:567278. doi: 10.3389/fpsyg.2020.567278 (PMC7854448; doi:10.3389/fpsyg.2020.567278)
Supplement: Supplementary file 1 [file Table_1.docx]

Supplementary Table 1. Relationship between emotion regulation and driving behaviour at baseline in the whole sample

| **Emotion regulation** | **Driving behaviour** | | | | | | | | | | | |  |
| --- | --- | --- | --- | --- | --- | --- | --- | --- | --- | --- | --- | --- | --- |
|  | Average Speed (km/h) | Variance speed (km/h) | Average Speed risk situations (km/h) | Variance speed risk situations (km/h) | Average exceeded speed limits (km/h) | Variance exceeded speed limits (km/h) | Length of time spent exceeding speed (sec) | Mean Throttle (%) | Front Brake (kg) | Variance Steering wheel (rad) | Accidents (sum) | Average Evaluation Risk situations (1-4) | |
| DERS |  |  |  |  |  |  |  |  |  |  |  |  | |
| Lack of emotional awareness | 0.267* | 0.184 | 0.246* | 0.215* | 0.253* | 0.220* | 0.196 | 0.092 | 0.039 | 0.211* | 0.215* | -0.233* | |
| Impulse control difficulties | 0.215* | 0.165 | 0.288** | 0.226* | 0.200 | 0.161 | 0.290*** | 0.126 | 0.055 | 0.231* | 0.097 | -0.335* | |
| Non acceptance of emotional response | 0.056 | 0.057 | 0.090 | 0.087 | 0.010 | 0.017 | 0.091 | -0.014 | 0.036 | 0.149 | 0.032 | -0.175 | |
| Difficulties engaging in goal directed behaviour | 0.124 | 0.095 | 0.173 | 0.118 | 0.172 | 0.141 | 0.164 | 0.118 | -0.053 | 0.107 | 0.066 | -0.120 | |
| Lack of emotional clarity | 0.262* | 0.199 | 0.261* | 0.203 | 0.237* | 0.232* | 0.234* | 0.171 | -0.037 | 0.232* | 0.296*** | -0.247*** | |
| Limited access to emotion regulation strategies | 0.121 | 0.137 | 0.216* | 0.178 | 0.162 | 0.214* | 0.214* | 0.126 | 0.132 | 0.195 | 0.248* | -0.329**** | |
| Total scores | 0.228* | 0.188 | 0.291*** | 0.235* | 0.230* | 0.224* | 0.275** | 0.140 | 0.054 | 0.258* | 0.190 | -0.342**** | |
|  |  |  |  |  |  |  |  |  |  |  |  |  | |
| CERQ - positve ER startegies | | | | | | | | | | | | | |
| Acceptance | -0.215* | -0.291*** | -0.145 | -0.278** | -0.242* | -0.223* | -0.221* | -0.189 | -0.170 | -0.131 | -0.147 | 0.181 | |
| Putting into perspective | -0.329*** | -0.379**** | -0.265* | -0.326*** | -0.355**** | -0.340**** | -0.341**** | -0.357**** | -0.199 | -0.330*** | -0.277** | 0.267* | |
| Refocus on planning | -0.266* | -0.236* | -0.256* | -0.289** | -0.239* | -0.245* | -0.255* | -0.160 | -0.164 | -0.242* | -0.110 | 0.234* | |
| Positive refocusing | -0.112 | -0.138 | -0.081 | -0.156 | -0.150 | -0.143 | -0.134 | -0.095 | -0.179 | -0.104 | -0.025 | 0.148 | |
| Positive Reappraisal | -0.318*** | -0.352**** | -0.318*** | -0.358**** | -0.347**** | -0.365**** | -0.352**** | -0.302*** | -0.181 | -0.282** | -0.183 | 0.298*** | |
| Total score | -0.334*** | -0.375**** | -0.289** | -0.376**** | -0.360**** | -0.356**** | -0.352**** | -0.300*** | -0.239* | -0.298*** | -0.195 | 0.304*** | |
|  |  |  |  |  |  |  |  |  |  |  |  |  | |
| CERQ - negative ER startegies | | | | | | | | | | | | | |
| Catastrophizing | 0.049 | 0.033 | 0.170 | 0.085 | 0.081 | 0.095 | 0.124 | 0.008 | 0.081 | 0.043 | 0.116 | -0.232* | |
| Other blame | -0.008 | -0.029 | 0.060 | -0.015 | -0.009 | 0.010 | -0.050 | -0.118 | -0.164 | -0.077 | 0.228* | 0.023 | |
| Rumination | -0.288** | -0.297*** | -0.212* | -0.330*** | -0.284** | -0.219* | -0.274** | -0.169 | -0.102 | -0.186 | -0.137 | 0.164 | |
| Self blame | -0.090 | 0.006 | -0.011 | 0.039 | -0.046 | 0.025 | 0.038 | 0.012 | -0.077 | -0.061 | -0.190 | 0.019 | |
| Total scores | -0.130 | -0.113 | -0.005 | -0.091 | -0.101 | -0.039 | -0.064 | -0.096 | -0.085 | -0.102 | -0.025 | -0.012 | |

Note: **** p < 0.001, *** p < 0.005, ** p < 0.01, * p < 0.05; Rear Brake Index did not show any significant relationship. Partial correlation coefficient reported represents Pearson’s r, except for accident rates where Spearman’s rho is reported.
